# Heliox ventilation promotes pulmonary circulation and cardiac function in elderly hypertensive patients: a randomized controlled trial

**DOI:** 10.4103/mgr.MEDGASRES-D-25-00090

**Published:** 2026-01-23

**Authors:** Lili Zhou, Lihui Chen, Jing Lin, Mingkai Zhuang, Jinzhang Zhuo, Hui Zhang, Qinyong Weng

**Affiliations:** 1 Department of Geriatrics, Fujian Medical University Union Hospital, Fuzhou, Fujian Province, China; 2 Department of Critical Care Medicine, Fujian Medical University Union Hospital, Fuzhou, Fujian Province, China; 3 Department of Laboratory Medicine, Pingtan Comprehensive Experimental Area Hospital, Fuzhou, Fujian Province, China; 4 Department of Cardiovascular Surgery, Fujian Medical University Union Hospital, Fuzhou, Fujian Province, China; 5 Department of Digestive Medicine, Fujian Medical University Union Hospital, Fuzhou, Fujian Province, China

**Keywords:** airway pressures, cardiac function, caveolin-1, central venous oxygen saturation, helium–oxygen inhalation, nitric oxide, nitrogen–oxygen ventilation, older patients with hypertension, positive-pressure ventilation, pulmonary circulation

## Abstract

Previous studies have indicated that helium–oxygen mixture (heliox) ventilation could improve blood pressure and microcirculation in elderly hypertensive patients. To explore the advantages of heliox ventilation over conventional nitrogen–oxygen ventilation, a randomized controlled study was conducted from October 2020 to January 2023 in the Intensive Care Unit of Fujian Medical University Union Hospital and included 40 elderly hypertensive patients requiring invasive mechanical ventilation. These patients were randomly assigned to two groups: the heliox ventilation group (*n* = 20), which received a closed heliox ventilation protocol for 3 hours, and the nitrogen–oxygen ventilation group (*n* = 20), which received conventional nitrogen–oxygen ventilation. Compared with the nitrogen-oxygen group, the heliox group demonstrated significantly lower central venous pressure and higher central venous oxygen saturation, indicating increased cardiac output and elevated plasma nitric oxide. Moreover, in the heliox group, the change in plasma caveolin-1 was essentially identical to that in nitric oxide. However, there was no significant difference in endothelin-1 levels between the two groups. These findings indicate that heliox ventilation enhances cardiac function in elderly hypertensive patients by improving pulmonary circulation through increased pulmonary vasodilation. The trial was also registered in the Chinese Clinical Trial Registry (registration No. ChiCTR2100043945) on March 6, 2021.

## Graphical abstract

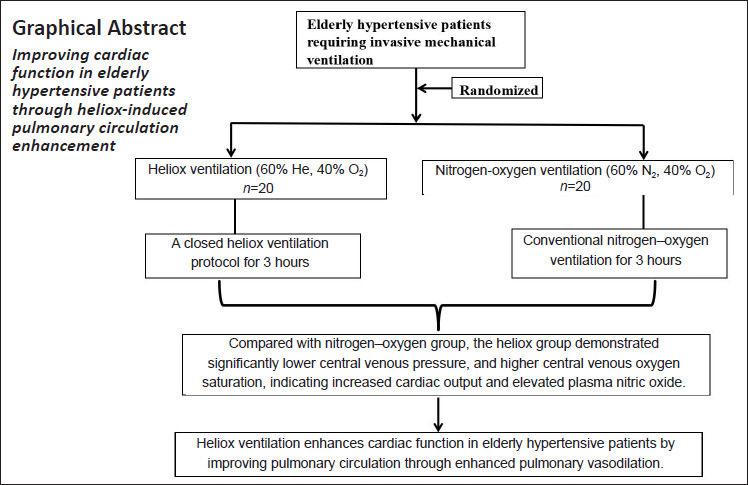

## Introduction

Around the globe, the prevalence of essential hypertension among elderly patients has rapidly increased.^1^ Recent statistics from the National Health and Nutrition Examination Survey show that hypertension impacts approximately 70% of older adults.^2^ In aging individuals with hypertension, the elastic arteries gradually lose their elasticity and become rigid. This results in a reduction in their compliance and impairs their capacity to effectively buffer the pressure fluctuations that occur during each cardiac cycle.^3^ When the cardiac output of elderly hypertensive patients decreases, they experience immediate decreases in blood pressure.

Traditional methods such as pulmonary artery catheterization and pulse indicator continuous cardiac output are invasive and costly.^4^ Echocardiography is non-invasive, but the results can vary. This is because of factors such as how the patient is positioned, interference from gases used in mechanical ventilation, and differences in the operator’s skills.^5^ These problems mean that these methods are not widely used in clinical settings. Another important part of hemodynamic stability is achieving a balance between the oxygen supply and consumption. This is checked at the bedside by looking at central venous oxygen saturation (ScvO_2_). The ScvO_2_ level decreases when the oxygen supply is insufficient. This can occur if the cardiac output is reduced, the hemoglobin level is low, or the arterial oxygen saturation is low. It can also occur when the body needs more oxygen, such as when there is a fever, the patient is agitated, they are shivering, or they have a high metabolism.^6^ Therefore, when the hemoglobin concentration, arterial oxygen saturation, and metabolic demand remain stable, ScvO_2_ is positively related to cardiac output. Some recent research has shown that measuring ScvO_2_ can reveal how well the left ventricle is ejecting blood. It is simple, reliable, and has considerable potential for use in clinical situations.^7^,^8^,^9^ Therefore, in this study, ScvO_2_ was used as an indicator of left ventricular ejection.

In elderly patients with hypertension, their hemodynamic situation is especially susceptible to positive-pressure ventilation. Earlier studies by our team revealed that applying a positive end-expiratory pressure (PEEP) greater than 4 cm H_2_O (1 cmH_2_O ≈ 98 Pa) to hypertensive patients resulted in a decrease in blood pressure and ScvO_2_. Moreover, there was an increase in heart rate, indicating an reduction in cardiac output.^10^ Older hypertensive patients who have acute respiratory distress syndrome or other lung conditions need high levels of PEEP. This is to prevent alveolar collapse, reduce the accumulation of alveolar fluid as much as possible, and increase lung compliance.^11^ However, it is quite a challenge to handle the adverse hemodynamic effects associated with high PEEP. There is not much research on lessening these impacts. Therefore, it is essential to explore the possibility of using heliox ventilation to lessen the hemodynamic disturbances caused by positive-pressure ventilation in elderly hypertensive patients.

The helium–oxygen mixture (heliox), which is made up of helium and oxygen, has an obviously lower density than air. This means that it can reduce airway resistance and increase the efficiency of gas flow.^12^ Truebel et al.^13^ showed that healthy volunteers who had heliox ventilation had fewer changes in blood pressure caused by mechanical ventilation. As a result, their hemodynamic stability improved. Our previous research also confirmed that heliox ventilation could improve blood pressure and microcirculatory function in older hypertensive patients. It might even address some of the problems that are part of conventional nitrogen–oxygen ventilation.^14^ We believe that because of its low density and high diffusivity, heliox can decrease turbulent airflow and airway resistance. This then leads to a reduction in the intrinsic PEEP and lung hyperinflation. Therefore, this process may maintain venous return and pulmonary vascular resistance and, in turn, improve overall hemodynamic stability.

Nevertheless, it is still not clear whether heliox ventilation can increase cardiac performance by enhancing pulmonary perfusion and thus has a positive impact on both macrocirculation and microcirculation, especially in elderly hypertensive patients. Previously, our research team used a rabbit acute lung injury model and reported that heliox ventilation could reduce the amount of inflammatory exudates in pulmonary vessels and alveolar spaces, which subsequently led to an improvement in lung tissue perfusion.^15^,^16^ We hypothesized that heliox might further enhance hemodynamic stability by improving pulmonary circulation. Additionally, recent studies have shown that heliox could promote organ perfusion through the nitric oxide (NO) signaling pathway,^17^,^18^ and it might also involve an increase in circulating caveolin-1 (Cav-1).^19^,^20^ Therefore, we propose that heliox ventilation might stabilize hemodynamics in elderly hypertensive patients who are receiving mechanical ventilation by adjusting pulmonary vasoactive mediators and protecting the function of the pulmonary vascular endothelium. However, more studies are needed to confirm these mechanisms.

Although there could be certain clinical advantages, the extensive use of heliox is still limited. This is because it has a high cost and leaks quickly from normal ventilation circuits. The quick leakage is due to its high diffusivity. To address this real limitation, we developed an inventive, tightly sealed ventilator circuit. This circuit is designed to recycle helium gas in an effective way, which can greatly reduce treatment costs and make it easier to be widely used in clinical settings.

The aim of the present study was to determine whether heliox ventilation, compared with conventional nitrogen–oxygen ventilation, could improve cardiac function by increasing pulmonary vasodilatory factors, thus enhancing pulmonary circulation and lessening the adverse hemodynamic effects of mechanical ventilation in elderly hypertensive patients. In addition, efforts should be made to expand the clinical application of this innovative, tightly sealed ventilator circuit.

## Methods

### Study design

The subjects received either heliox or nitrogen–oxygen ventilation for 3 hours according to the groups to which they were assigned. During this time, the tidal volume and PEEP were maintained constantly. The systolic blood pressure (SBP), diastolic blood pressure (DBP), mean arterial blood pressure (MABP), central venous pressure (CVP), ScvO_2_, heart rate, airway pressure, and plasma lactic acid (Lac) levels were recorded every hour. The plasma NO, endothelin-1 (ET-1), and Cav-1 concentrations were measured at the starting point (0 hours) and at 3, 9, and 24 hours after the intervention. This was done to observe the changes over time. The investigators and data collectors did not know which ventilation method the patients were using because both groups were given mechanical ventilation. To guarantee the safety of the patients, the study protocol stated that the study should be stopped if the blood pressure dropped by more than 20% compared with the starting level, if the peripheral oxygen saturation (SpO_2_) fell below 95%, or if there was ventilator–patient asynchrony. The study was approved by the Ethical Committee of Fujian Medical University Union Hospital (approval No. 2020WSJK002) on August 20, 2020. The trial was also registered in the Chinese Clinical Trial Registry (registration No. ChiCTR2100043945) on March 6, 2021. The CONsolidated Standards Of Reporting Trials (CONSORT) 2010 guidelines were strictly followed when reporting this study.^21^ All the study procedures adhered strictly to the ethical standards outlined by the institutional/national research committee and the *Helsinki Declaration* (1964), including subsequent amendments and similar ethical guidelines.

### Subjects

This randomized controlled trial happened from October 2020 to January 2023 in the Intensive Care Unit of Fujian Medical University Union Hospital. A total of 40 elderly patients were included in the experiment. Each of them either provided written informed consent on their own or was given through their legally authorized representatives. The inclusion criteria were as follows: aged 65–95 years, a diagnosis of hypertension, respiratory failure because of pneumonia, invasive mechanical ventilation for more than 24 hours, and a stable SpO_2_ exceeding 95% for at least 30 minutes while keeping the oxygen concentration at 40%. Patients were excluded if they had secondary hypertension, if they were in shock, if they had arrhythmia, if they had severe heart failure (New York Heart Association class^22^ III–IV), if they needed vasoactive medications, or if they were diagnosed with pulmonary malignancies.

**Figure 1:**
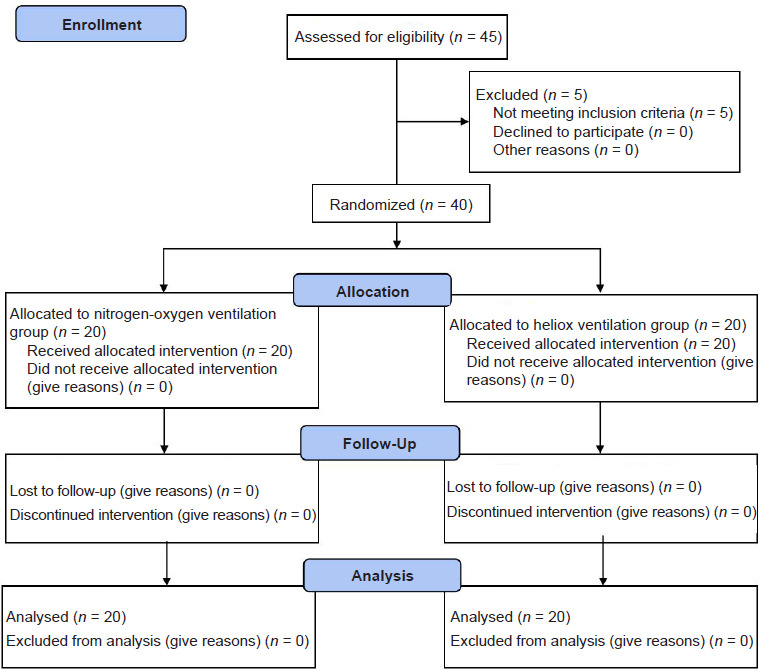
CONsolidated Standards Of Reporting Trials (CONSORT) flow diagram. These patients were randomly assigned to two groups: the heliox ventilation group (*n* = 20) and the nitrogen–oxygen ventilation group (*n* = 20).

The participants were randomly divided into two groups. The nitrogen–oxygen ventilation group included 20 participants, and the heliox ventilation group included 20 participants (**Figure 1**). The random division was performed through computer-generated numbers ranging from 1 to 40. The assignments were then sealed inside envelopes and only opened once the patients had been enrolled in the study.

### Heliox ventilation

For every patient who was enrolled, a Vela VCV mode ventilator (Vyaire Medical, Chicago, IL, USA) was used to offer mechanical ventilation. The settings of the ventilator were as follows: the tidal volume was set at 8 mL/kg, the optimal PEEP ranged from 5 to 8 cmH_2_O, which was determined based on achieving the best oxygenation, the inspiratory to expiratory ratio was 1:2, and the respiratory rate was 18 breaths per minute to maintain sufficient oxygenation and ensure that carbon dioxide could be removed properly. Patients whose SpO_2_ remained above 95% when the oxygen concentration was 40% were included in this study. Continuous invasive arterial blood pressure monitoring was carried out through radial artery catheterization. Moreover, the CVP and ScvO_2_ were measured by using a subclavian venous catheter. The airway secretions were cleared on a regular basis, and the patients were placed in a 30° semi-recumbent position in a comfortable way. The intravenous infusion rates were maintained at 40–50 drops per minute. Midazolam (0.05 mg/kg/h; Jiangsu Nhwa Pharmaceutical Co., Ltd., Jiangsu, China) and remifentanil (0.05–0.08 µg/kg/min; Yichang Humanwell Pharmaceutical Co., Ltd., Yichang, China) were used for sedation and analgesia. This was to reduce spontaneous breathing as much as possible and to keep the Richmond Agitation-Sedation Scale scores^23^ between –2 and –3 and the Critical Care Pain Observation Tool scores^24^ between 0 and 2.

The operational steps for closed-circuit heliox ventilation were as follows. First, we adjusted the airflow of the anesthetic machine (Dräger Medical, Lübeck, Germany) to zero. Then, we connected the helium gas through the nitrous oxide pathway. Before starting the study, we connected the anesthetic machine to the simulated lungs and ventilated them with pure oxygen for approximately 30 minutes. During this process, we continuously introduced helium gas into the circuit to eliminate residual nitrogen. We also connected an oxygen concentration monitor to the gas delivery endpoint of the anesthetic machine and ensured that the total flow of helium and oxygen gas remained constant. We continuously adjusted the flow rates of helium and oxygen to keep the oxygen concentration stable at 40%. In the end, we connected the anesthetic machine to the patient. In this case, the body consumed some of the oxygen, and the exhaled helium was recycled back to the respiratory circuit for the next breath. That is, the recycling of helium was achieved.

### Measurements of lung function and hemodynamics

The CVP, radial arterial blood pressure, heart rate, and SpO_2_ were measured via a multiparameter cardiac monitor (GEcicpro/8000i, Madison, WI, USA). The central venous blood (approximately 1.0 mL) for blood gas analysis was analyzed using an i-stat300 blood gas analyzer (Abbott, Chicago, IL, USA) to evaluate ScvO_2_ and Lac. The ventilator or anesthetic machine continuously recorded the peak airway pressure (Ppeak), plateau airway pressure (Pplat), driving pressure (ΔP), tidal volume, and minute ventilation volume. We recorded all the above parameters at the beginning (0 hours) and then again after 1, 2, and 3 hours of ventilation. We collected 2.0 mL of arterial blood samples at 0, 3, 9, and 24 hours from the start of ventilation to check the levels of plasma NO, ET-1, and Cav-1. We measured plasma NO (NO determination kit, A013-2-1, Nanjing Jiancheng Bioengineering Institute, Nanjing, China) by using the nitrate reduction method, and we quantified ET-1 (human ET-1 test kit, MM-14267H1, Jiangsu Meimian Industrial Co., Ltd., Jiangsu, China) and Cav-1 (human Cav-1 test kit, MM-1093H1, Jiangsu Meimian Industrial Co., Ltd.) by using enzyme-linked immunosorbent assay techniques.

The primary outcome was ScvO_2_. Moreover, radial arterial blood pressure, CVP, heart rate, airway pressure, and pulmonary vasoactive factors were regarded as secondary outcomes.

### Evaluation criteria for hypertension

Hypertension was characterized by having an SBP that was equal to or greater than 140 mmHg and/or a DBP that was equal to or greater than 90 mmHg. This measurement was taken during mechanical ventilation when there was sufficient sedation and no issues of ventilator–patient asynchrony. Another situation was considered hypertension if the patient self-reported that they regularly used antihypertensive medication within 2 weeks.^25^

### Sample size calculation

We used NCSS PASS statistical software (version 15.0; NCSS, LLC, Kaysville, UT, USA) to calculate the sample size. The main outcome measure we focused on was ScvO_2_. We calculated the sample size by using the repeated measures analysis of variance, where we set α to 0.05 and the statistical power (1 – β) to 0.8. If we assume a 1:1 allocation, then we would need a minimum of 36 patients, that is, 18 patients in each group. When we consider the dropout rate, which is approximately 10%, at least 40 patients are needed, with 20 patients in each group.

### Statistical analysis

SPSS version 24.0 (IBM Corporation, Armonk, NY, USA) was used for conducting the statistical analyses. All the continuous data are presented as the mean ± standard deviation (SD) or median (interquartile range), which were determined via normality assessment via the Shapiro–Wilk test. For the comparisons between groups, when the data were normally distributed, we used Student’s *t*-test or repeated measures analysis of variance. The Bonferroni method was applied for further pairwise comparisons. If the data were not normally distributed, the Mann–Whitney *U* or Kruskal–Wallis test was used. The chi-square test or Fisher’s exact test was used to analyze the categorical variables. Pearson’s correlation analysis was carried out to assess the correlations. We adjusted for potential confounding factors via multiple linear or logistic regression analyses. If the *P* value was less than 0.05, it was regarded as statistically significant.

## Results

### Participant characteristics

We finally enrolled 40 patients, including 20 patients in the nitrogen–oxygen ventilation group and 20 patients in the heliox ventilation group, actually meeting the sample size criteria. This new and creative study made use of a sealed breathing circuit. This circuit could significantly increase the utilization of helium. Notably, with this circuit, a single 40 L cylinder could support ventilation for 10 patients instead of just one patient.

The descriptive data of the study population are summarized in **Table 1**. No significant differences in sex, age, serum creatinine, uric acid, triglyceride, total cholesterol, low-density lipoprotein cholesterol, fasting plasma glucose, hemoglobin, or body temperature were detected between the two groups (all *P* > 0.05). Baseline hemodynamic parameters, including SBP, DBP, MABP, CVP, Lac, and SpO_2_, also showed no statistically significant differences between the two groups (*P* > 0.05). Additionally, baseline comorbidities did not significantly differ: the nitrogen–oxygen ventilation group included 2 patients with coronary atherosclerotic heart disease, 3 patients with hypertensive heart disease, and 15 patients without heart disease, whereas the heliox ventilation group included 1 patient with coronary atherosclerotic heart disease, 2 patients with chronic pulmonary heart disease, 4 patients with hypertensive heart disease, and 13 patients without heart disease (Fisher’s exact test: *P* = 0.634). Similarly, baseline pulmonary diseases did not differ significantly: the nitrogen–oxygen ventilation group included 2 patients with chronic obstructive pulmonary disease, 2 with bronchiectasis, and 16 patients without lung disease; the heliox ventilation group included 4 patients with chronic obstructive pulmonary disease and 16 without pulmonary disease (Fisher’s exact test: *P* = 0.415).

**Table 1:** Baseline characteristics

	Nitrogen–oxygen ventilation group (*n*=20)	Heliox ventilation group (*n*=20)	*P* value
Sex (male/female) (*n*)	17/3	12/8	0.155
Age (yr)	77.25±8.59	78.30±7.22	0.678
SBP (mmHg)	144.15±22.68	139.70±15.26	0.472
DBP (mmHg)	63.40±12.47	59.35±10.18	0.268
MABP (mmHg)	90.32±14.23	86.13±10.46	0.297
Scr (μM)	66.50 (40.75, 93.50)	70.50 (54.25, 111.25)	0.394
UA (μM)	230.45±130.82	294.50±132.45	0.132
TG (mM)	0.90 (0.74, 1.72)	0.75 (0.54, 1.07)	0.137
TC (mM)	3.96 (2.54, 4.59)	2.89 (2.42, 4.26)	0.317
LDL-C (mM)	2.47 (1.63, 3.20)	2.00 (1.40, 2.88)	0.350
FPG (mM)	7.49 (5.82, 13.45)	7.55 (6.29, 10.05)	0.552
HB (g/L)	104.20±19.08	94.25±19.24	0.109
T (°C)	36.84±0.60	36.72±0.55	0.532
CVP (mmHg)	8.83±0.65	9.14±0.61	0.135
Lac (mM)	1.40 (1.20, 2.18)	1.25 (1.03, 1.65)	0.271
SpO_2_ (%)	99.00 (98.00, 100.00)	98.00 (96.00, 99.75)	0.055

Data are shown as the mean ± standard deviation or median (interquartile). *P < 0.05 (Fisher’s exact test, Student’s *t*-test or the Mann–Whitney *U* test). CVP: Central venous pressure; DBP: diastolic blood pressure; FPG: fasting plasma glucose; HB: hemoglobin; Lac: plasma lactic acid; LDL-C: low-density lipoprotein cholesterol; MABP: mean arterial blood pressure; SBP: systolic blood pressure; Scr: serum creatinine; SpO_2_: peripheral oxygen saturation; T: body temperature; TC: total cholesterol; TG: triglyceride; UA: uric acid.

Significant differences were observed in the mean SBP, DBP, and MABP across time points (corrected *P* < 0.05), as were significant interactions between ventilation mode and treatment duration (*P* < 0.05), indicating an impact of ventilation mode on these outcomes. No significant differences were observed in the mean SBP, DBP, or MABP between the two groups (*P* > 0.05; **Additional Tables 1–3** and **Additional Figure 1**).

**Additional Table 1:** Variance of repeated measurement of systolic blood pressure

Variation	Sum of squares of deviation	Degrees of freedom	Mean square	*F*	*P*
Time	1581.619	3	527.206	16.801	<0.001
Group	3431.756	1	3431.756	2.681	0.110
Time×group	2597.919	3	865.973	27.597	<0.001

**Additional Table 2:** Variance of repeated measurement of diastolic blood pressure

Variation	Sum of squares of deviation	Degrees of freedom	Mean square	*F*	*P*
Time	114.150	3	38.050	5.754	0.001
Group	44.100	1	44.100	0.102	0.751
Time×group	363.050	3	121.017	18.302	<0.001

**Additional Table 3:** Variance of repeated measurement of mean arterial blood pressure

Variation	Sum of squares of deviation	Degrees of freedom	Mean square	*F*	*P*
Time	414.647	3	138.216	13.912	<0.001
Group	573.806	1	573.806	1.124	0.296
Time×group	877.158	3	292.386	29.429	<0.001

The tidal volume (Kruskal–Wallis tests: *P* = 0.746 in the nitrogen–oxygen ventilation group and *P* = 0.933 in the heliox ventilation group) and minute ventilation volume (Kruskal–Wallis tests: *P* = 0.993 in the nitrogen–oxygen ventilation group and *P* = 0.954 in the heliox ventilation group) remained unchanged throughout ventilation in both groups (**Additional Table 4**). Repeated-measures analysis of variance revealed significant changes over time for Ppeak, Pplat, and ΔP (corrected *P* < 0.05) and significant interactions between ventilation type and time (*P* < 0.05), suggesting an impact of ventilation mode on airway pressure. However, no significant differences in Ppeak, Pplat and ΔP were observed between the two groups (all *P* > 0.05; **Additional Tables 5–7** and **Additional Figure 2**).

**Additional Table 4:** Effect of different ventilation modes on hemodynamics

		Ventilation time (h)			
		0	1	2	3
SBP (mmHg)	Nitrogen-oxygen ventilation group	144.15±22.68	129.65±21.77	128.80±19.01	125.55±22.10
	Heliox ventilation group	139.70±15.26	141.45±15.23	142.45±14.95	141.60±14.91
DBP (mmHg)	Nitrogen-oxygen ventilation group	63.40±12.47	58.45±11.42	58.55±11.69	57.30±10.72
	Heliox ventilation group	59.35±10.18	60.95±9.08	60.55±8.99	61.05±10.06
MABP (mmHg)	Nitrogen-oxygen ventilation group	90.32±14.23	82.18±13.51	81.97±12.20	80.05±12.63
	Heliox ventilation group	86.13±10.46	87.78±9.40	87.85±9.32	87.90±10.12
CVP (mmHg)	Nitrogen-oxygen ventilation group	8.83±0.65	9.35±0.68	9.29±0.68	9.35±0.82
	Heliox ventilation group	9.14±0.61	8.21±0.48	8.13±0.55	8.12±0.51
ScvO_2_ (%)	Nitrogen-oxygen ventilation group	74.25±4.24	67.40±3.42	68.20±3.09	67.60±3.49
	Heliox ventilation group	67.05±3.52	73.80±4.26	73.85±4.57	74.30±4.61
HR (beats/min)	Nitrogen-oxygen ventilation group	75.20±16.56	81.45±17.41	81.45±17.84	81.45±17.75
	Heliox ventilation group	67.65±13.89	67.55±13.32	68.90±13.10	68.40±12.93
Lac (mM)	Nitrogen-oxygen ventilation group	1.40 (1.20, 2.18)	1.45 (1.30, 2.15)	1.45 (1.20, 2.28)	1.30 (1.20, 2.03)
	Heliox ventilation group	1.25 (1.03, 1.65)	1.30 (1.00, 1.50)	1.30 (1.03, 1.65)	1.20 (1.00, 1.55)
Ppeak (cmH_2_O)	Nitrogen-oxygen ventilation group	19.75±1.94	22.35±2.13	22.30±2.11	22.15±2.16
	Heliox ventilation group	28.00±5.07	22.80±4.47	21.55±4.14	21.25±3.75
Pplat (cmH_2_O)	Nitrogen-oxygen ventilation group	16.50±1.88	18.50±1.70	18.55±1.82	18.50±2.04
	Heliox ventilation group	23.20±3.81	19.15±3.90	17.85±3.84	17.55±3.69
ΔP (cmH_2_O)	Nitrogen-oxygen ventilation group	10.50±1.88	12.50±1.70	12.55±1.82	12.50±2.04
	Heliox ventilation group	17.20±3.81	13.15±3.90	11.85±3.84	11.55±3.69
VT (mL)	Nitrogen-oxygen ventilation group	499.5 (480, 521.75)	499.5 (482, 523)	500 (481, 523)	501 (482.25, 523)
	Heliox ventilation group	478 (400, 518.5)	480 (402.25, 518.25)	479.5 (405, 520)	477.5 (403, 520.25)
MV (L/min)	Nitrogen-oxygen ventilation group	8.93±0.93	8.83±0.93	8.81±0.82	8.80±0.78
	Heliox ventilation group	8.12±1.37	8.19±1.31	8.24±1.32	8.21±1.37
SpO_2_ (%)	Nitrogen-oxygen ventilation group	144.15±22.68	129.65±21.77	128.80±19.01	125.55±22.10
	Heliox ventilation group	139.70±15.26	141.45±15.23	142.45±14.95	141.60±14.91

Data are shown as mean ± SD or median (interquartile). ΔP: Driving pressure; CVP: central venous pressure; DBP: diastolic blood pressure; HR: heart rate; Lac: plasma lactic acid; MABP: mean arterial blood pressure; MV: minute ventilation volume; Ppeak: peak airway pressure; Pplat: platform airway pressure; SBP: systolic blood pressure; ScvO_2_: central venous oxygen saturation; SpO_2_: peripheral oxygen saturation; VT: tidal volume.

**Additional Table 5:** Variance of repeated measurement of peak airway pressure

Variation	Sum of squares of deviation	Degrees of freedom	Mean square	*F*	*P*
Time	114.619	1.766	64.915	25.248	< 0.001
Group	124.256	1	124.256	2.917	0.096
Time×group	572.119	1.766	324.021	126.023	< 0.001

**Additional Table 6:** Variance of repeated measurement of platform airway pressure

Variation	Sum of squares of deviation	Degrees of freedom	Mean square	*F*	*P*
Time	81.650	2.023	40.366	19.697	< 0.001
Group	81.225	1	81.225	2.552	0.118
Time×group	385.825	2.023	190.742	93.073	< 0.001

**Additional Table 7:** Variance of repeated measurement of driving pressure

Variation	Sum of squares of deviation	Degrees of freedom	Mean square	*F*	*P*
Time	81.650	2.023	40.366	19.697	< 0.001
Group	81.225	1	81.225	2.552	0.118
Time×group	385.825	2.023	190.742	93.073	< 0.001

### Heliox ventilation improves cardiac function in elderly hypertensive patients

**Figure 2:**
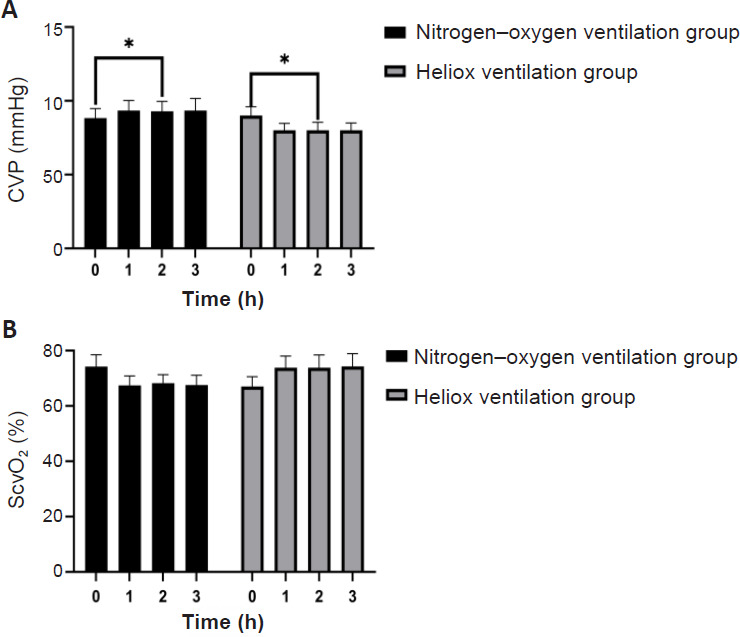
Effects of different ventilation modes on central venous pressure (CVP, A) and central venous oxygen saturation (ScvO_2_, B). The data are shown as mean ± SD. *P < 0.05, *vs*. 0 hours (repeated measures analysis of variance followed by the Bonferroni method).

Repeated-measures analysis of variance revealed significant differences in CVP across time points (corrected *P* < 0.05) and a significant interaction effect between ventilation type and time (*P* < 0.05), indicating that the ventilation mode influenced CVP. The mean CVP differed significantly between the two groups (*P* < 0.05; **Figure 2** and **Additional Table 8**). Ventilation duration was positively correlated with CVP in the nitrogen–oxygen ventilation group (*r* = 0.232, *P* = 0.039), even after controlling for confounding variables. In contrast, ventilation duration was negatively correlated with CVP in the heliox ventilation group (*r* = –0.516, *P* < 0.001), and this negative correlation persisted after adjustment for confounders (**Additional Table 9**). Repeated-measures analysis of variance revealed that ScvO_2_ did not significantly vary over time within groups (corrected *P* > 0.05). However, a significant interaction between ventilation type and time was found (corrected *P* < 0.05), indicating that ventilation type influenced the ScvO_2_ measurements over time. The mean ScvO_2_ differed significantly between the two groups (*P* < 0.05; **Figure 2** and **Additional Table 10**). Ventilation duration was negatively correlated with ScvO_2_ in the nitrogen–oxygen ventilation group (*r* = –0.476, *P* < 0.001), which persisted after controlling for confounding variables. Conversely, ventilation duration was positively correlated with ScvO_2_ in the heliox ventilation group (*r* = 0.475, *P* = 0.001), remaining significant after controlling for confounders (**Additional Table 11**).

**Additional Table 8:** Variance of repeated measurement of central venous pressure

Variation	Sum of squares of deviation	Degrees of freedom	Mean square	*F*	*P*
Time	1.869	1.329	1.406	5.874	0.012
Group	25.921	1	25.921	20.508	< 0.001
Time×group	16.475	1.329	12.394	51.794	< 0.001

**Additional Table 9:** Multiple linear regression analysis of central venous pressure and ventilation time

	Model 1	Model 2	Model 3
Nitrogen-oxygen ventilation group			
*R*	0.244	0.295	0.367
*P*	0.031	0.013	0.002
Heliox ventilation group			
*R*	−0.530	−0.626	−0.634
*P*	< 0.001	< 0.001	< 0.001

Model 1: Adjustment for age and gender; Model 2: Adjustment for age, gender, basic heart and pulmonary disease, triglyceride, total cholesterol, low density lipoprotein cholesterin, fasting plasma glucose, serum creatinine and uric acid; Model 3: Adjustment for hemoglobin, body temperature, minute ventilation volume and peripheral oxygen saturation on the basis of Model 2.

**Additional Table 10:** Variance of repeated measurement of central venous oxygen saturation

Variation	Sum of squares of deviation	Degrees of freedom	Mean square	*F*	*P*
Time	5.419	1.534	3.532	0.904	0.387
Group	333.506	1	333.506	5.951	0.019
Time×group	1362.619	1.534	888.071	227.390	< 0.001

**Additional Table 11:** Multiple linear regression analysis of central venous oxygen saturation and ventilation time

	Model 1	Model 2	Model 3
Nitrogen-oxygen ventilation group			
*r*	−0.482	−0.575	−0.605
*P*	< 0.001	< 0.001	< 0.001
Heliox ventilation group			
*r*	0.477	0.537	0.644
*P*	< 0.001	< 0.001	< 0.001

Model 1: Adjustment for age and gender; Model 2: adjustment for age, gender, basic heart and pulmonary disease, triglyceride, total cholesterol, low density lipoprotein cholesterin, fasting plasma glucose, serum creatinine and uric acid; Model 3: adjustment for hemoglobin, body temperature, minute ventilation volume and peripheral oxygen saturation on the basis of Model 2.

Heart rate varied significantly across time points (Greenhouse–Geisser correction, *P* < 0.05), and the interaction effect between ventilation type and time was significant (corrected *P* < 0.05). The overall mean heart rate significantly differed between the two groups (*P* < 0.05; **Additional Table 12**). Lac levels (Kruskal–Wallis tests: *P* = 0.914 in the nitrogen–oxygen ventilation group and *P* = 0.954 in the heliox ventilation group) and SpO_2_ levels (Kruskal–Wallis tests: *P* = 0.794 in the nitrogen–oxygen ventilation group and *P* = 0.849 in the heliox ventilation group) did not significantly change during ventilation in either group (**Additional Table 4**).

**Additional Table 12:** Variance of repeated measurement of heart rate

Variation	Sum of squares of deviation	Degrees of freedom	Mean square	*F*	*P*
Time	364.669	2.001	182.208	22.381	< 0.001
Group	5534.256	1	5534.256	5.866	0.020
Time×group	245.919	2.001	122.874	15.093	< 0.001

### Heliox ventilation promotes the secretion of pulmonary vasoactive substances in elderly hypertensive patients

**Figure 3:**
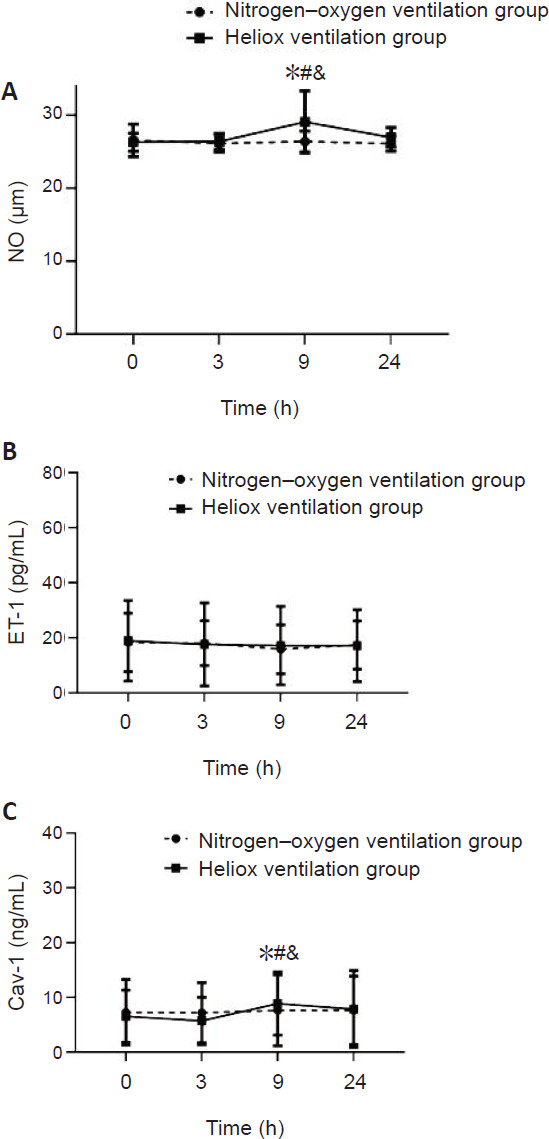
Effects of different ventilation modes on nitric oxide (NO, A), endothelin-1 (ET-1, B) and caveolin-1 (Cav-1, C) levels. The data are presented as mean and standard deviation. *P < 0.058, *vs*. 0 hours; ^#^*P* < 0.05, *vs*. 3 hours; ^&^*P* < 0.05, *vs*. 24 hours (repeated measures analysis of variance followed by the Bonferroni method).

The plasma NO, ET-1, and Cav-1 levels at different time points are summarized in **Table 2**. Repeated-measures analysis of variance revealed significant time-dependent changes in NO levels (corrected *P* < 0.05), with a significant interaction between ventilation type and time (corrected *P* < 0.05). The plasma NO concentrations were significantly greater in the heliox ventilation group than in the nitrogen–oxygen ventilation group (*P* < 0.05), increasing sharply after ventilation, peaking at 9 hours, and then declining thereafter (*P* < 0.05). Compared with the nitrogen–oxygen ventilation group, the heliox ventilation group presented more pronounced changes from 3–9 hours and 9–24 hours (*P* < 0.05). Other intervals revealed no significant differences (*P* > 0.05; **Figure 3** and **Additional Table 13**).

**Table 2:** Effects of different ventilation modes on plasma pulmonary vasoactive factor levels

		Ventilation time (h)			
		0	3	9	24
NO (µM)	Nitrogen–oxygen ventilation group	26.55±2.26	26.15±1.24	26.40±1.45	26.12±1.06
	Heliox ventilation group	26.32±1.23	26.41±1.12	29.03±4.28	26.97±1.32
ET-1 (pg/mL)	Nitrogen–oxygen ventilation group	20.65±8.59	20.45±8.07	20.43±8.31	20.23±7.22
	Heliox ventilation group	15.78±9.82	14.48±9.14	15.30±9.91	16.43±8.63
Cav-1 (ng/mL)	Nitrogen–oxygen ventilation group	11.00±6.48	10.59±5.78	12.39±6.06	12.04±6.09
	Heliox ventilation group	7.42±4.98	6.03±5.00	12.34±4.77	10.63±8.08

The data are shown as the mean ± standard deviation. Cav-1: Caveolin-1; ET-1: endothelin-1; NO: nitric oxide.

**Additional Table 13:** Variance of repeated measurement of nitric oxide

Variation	Sum of squares of deviation	Degrees of freedom	Mean square	*F*	*P*
Time	51.581	1.683	30.655	5.031	0.013
Group	30.857	1	30.857	5.039	0.031
Time×group	46.829	1.683	27.831	4.567	0.019

Repeated-measures analysis of variance indicated that ET-1 was not significantly different across time points (corrected *P* > 0.05) and that there was no significant interaction between ventilation mode and time (corrected *P* > 0.05), with no differences in the mean ET-1 between the two groups (*P* > 0.05; **Figure 3** and **Additional Table 14**).

**Additional Table 14:** Variance of repeated measurement of endothelin-1

Variation	Sum of squares of deviation	Degrees of freedom	Mean square	*F*	*P*
Time	18.159	2.410	7.534	0.895	0.429
Group	976.858	1	976.858	3.414	0.072
Time×group	23.866	2.410	9.902	1.176	0.319

Repeated-measures analysis of variance revealed that Cav-1 had no significant ventilation mode × time interaction effect (corrected *P* > 0.05), although a significant overall difference over time occurred (corrected *P* < 0.05). Cav-1 initially decreased, peaked at 9 hours, and then declined in both groups (**Figure 3** and **Additional Table 15**).

**Additional Table 15:** Variance of repeated measurement of caveolin-1

Variation	Sum of squares of deviation	Degrees of freedom	Mean square	*F*	*P*
Time	419.803	2.378	176.512	4.365	0.011
Group	230.523	1	230.523	4.874	0.033
Time×group	124.879	2.378	52.507	1.298	0.280

## Discussion

Heliox ventilation can significantly improve the stability of blood pressure and cardiac function among elderly hypertensive patients who are receiving mechanical ventilation with elevated PEEP. PEEP is quite effective in preventing alveolar collapse and enhancing oxygenation.^26^ However, it has an adverse impact on hemodynamics, especially in older hypertensive individuals. These patients usually have a reduction in vascular elasticity, and their cardiovascular adaptability is also compromised.^27^ The aim of this study was to make a direct comparison of the hemodynamic consequences between heliox ventilation and traditional nitrogen–oxygen ventilation. Specifically, it was used to assess whether heliox could lessen the negative cardiovascular effects that are associated with higher PEEP levels (5–8 cm H_2_O).^28^,^29^,^30^

The findings showed that when patients were on prolonged ventilation, those receiving the usual nitrogen–oxygen mixtures experienced obvious decreases in their blood pressure. This occurred because the increased pressures in the airways pushed up the intrathoracic pressures, which then disrupted the venous return.^28^,^29^,^30^ On the other hand, the blood pressure levels of patients who were treated with heliox ventilation remained stable throughout the ventilation period. A good effect of heliox was observed in the large decreases in CVP and airway pressure, along with an increase in ScvO_2_. These findings suggest that, compared with nitrogen–oxygen ventilation, heliox ventilation results in better venous return, and the pulmonary circulation works well. In the end, this helps improve cardiac function and keeps the hemodynamic parameters stable.^31^,^32^,^33^

Mechanically speaking, the beneficial cardiovascular effects of heliox can be partly attributed to its physical features. Specifically, compared with nitrogen–oxygen mixtures, helium–oxygen mixtures have a lower density and greater diffusion ability.^33^,^34^,^35^ These physical traits work to reduce airway turbulence. In doing so, they lower the intrinsic airway pressure and reduce pulmonary vascular resistance. Once ventilation improves with the help of heliox, the endogenous PEEP and intrathoracic pressure decrease. As a result, the CVP also decreases. This reduction in pulmonary resistance makes it easier for venous return to improve and boosts pulmonary blood flow. This process subsequently enhances cardiac performance and systemic hemodynamic stability.^36^,^37^,^38^ First, the resistance of the respiratory circuit of the anesthesia machine in the heliox ventilation group was greater than that of the ventilator used in the nitrogen–oxygen ventilation group. This led to the heliox ventilation group having higher initial airway pressures. However, as the ventilation time increased, the airway pressures in the heliox ventilation group decreased, whereas those in the nitrogen–oxygen ventilation group increased. By the end of the 3-hour ventilation period, the airway pressures in both groups were approximately the same. Even so, the heliox ventilation group still presented better hemodynamic stability than the nitrogen–oxygen ventilation group. This shows that there might be other mechanisms at play, not just the reduction in cardiopulmonary interactions during positive-pressure ventilation, that contribute to the beneficial effects of heliox. Therefore, it is worth examining this further.

In addition to these physical attributes, the study delved into the cellular mechanisms that are responsible for the cardiovascular enhancements caused by heliox. Notably, there were significant increases in the levels of plasma NO after heliox ventilation.^36^,^39^ NO, which is known for its powerful vasodilatory effects, plays a crucial role in adjusting pulmonary circulation.^36^ Moreover, the levels of plasma Cav-1 were found to be elevated. Previous studies have demonstrated that Cav-1 can regulate the activity of endothelial nitric oxide synthase.^39^,^40^,^41^ A study utilizing immunoprecipitation and structural domain localization techniques demonstrated that endothelial nitric oxide synthase directly binds to the Cav-1 scaffolding domain (amino acid residues 82–101), with more precise binding occurring at a 10-amino acid subsequence (amino acids 90–99; Kd = 49 nM). This mutual regulatory interaction between Cav-1 and endothelial nitric oxide synthase is crucial for controlling their expression and functional activity.^41^ Specifically, Cav-1 affects NO synthesis by directly interacting with endothelial nitric oxide synthase, and in this way, Cav-1 influences vascular tone and endothelial function.^39^,^40^ Earlier studies suggested that exposure to helium promotes the secretion of Cav-1, which might increase endothelial nitric oxide synthase activity and consequently lead to an increase in NO production.^19^,^41^,^42^,^43^ Therefore, the elevated levels of NO and Cav-1 observed in this study likely represent a vital cellular mechanism through which heliox ventilation improves pulmonary circulation and cardiac function.^44^,^45^,^46^

Technologically speaking, this research also dealt with the economic and practical challenges related to heliox ventilation that had previously emerged. The high diffusion rate of helium usually means it is costly, as it quickly exits conventional respiratory circuits.^47^,^48^,^49^ In this study, something innovative was done. A hermetic breathing circuit was used. This circuit could prolong the use of helium gas. Originally, helium could only be used from a 40 L cylinder for approximately 15 minutes. However, with this new circuit, it could be used for more than 30 hours. This large extension in how long helium can be used greatly reduces therapeutic costs. This, in turn, made it more likely for this to be used in clinics and made it more economically viable for hospitals to adopt it widely.^47^,^50^

Even with these somewhat encouraging findings, the study was not without its hitches. One major issue was that it was not possible to measure the dynamic pressures in the esophagus. Well, the patients simply refused to go along with that.^25^,^51^ If esophageal pressure could be measured, we could obtain a much clearer and more exact understanding of changes in thoracic pressure. That, in turn, would have helped us paint a better picture of the connection between the changes in airway pressure and the instability in hemodynamics. As the lung volume status is highly important for hemodynamic instability in patients ventilated with positive pressure, a sedative drug called midazolam and an analgesic drug called remifentanil were used to minimize spontaneous breathing, and the tidal volume was maintained relatively constant in this study. Moreover, prior to the study, there were no statistically significant differences in blood pressure, CVP, or Lac between the heliox ventilation group and the nitrogen–oxygen ventilation group. This was indicative of the similar initial hemodynamic status between the two groups. During this study, patients’ body position and infusion speed were controlled to ensure similar fluid intake. In addition, due to the limited patient source, the effects of heliox ventilation on hemodynamics in older hypertensive patients with mechanical ventilation should be confirmed in a large-sample multicenter clinical study.

Overall, this study shows that heliox ventilation is a better way to stabilize hemodynamic parameters and increase cardiac function in elderly hypertensive patients who need mechanical ventilation at high PEEP levels.^52^ It can improve pulmonary circulation by taking advantage of physical airway properties as well as the cellular mechanisms that involve NO and Cav-1. Therefore, heliox ventilation has much potential for clinical use. There are several benefits. For example, it can offer earlier chances for extubation, improve patient prognosis, shorten the length of hospital stay, and reduce overall healthcare expenses. It would be beneficial to perform future large-scale and multicenter clinical trials to confirm these findings and explain more clearly the underlying physiological and molecular pathways.^52^

Positive-pressure ventilation has many positive effects, but it can have a large effect on the hemodynamic situation of elderly patients who have hypertension. There is a closed heliox ventilation method that includes recycling helium. This technique might improve cardiac function and make hemodynamics more stable. Part of this improvement comes from increasing the factors that dilate the pulmonary vessels. As a result, it can increase pulmonary circulation in older hypertensive patients who are receiving mechanical ventilation. All of this can be done with treatment costs that are much lower.

## Additional files:

***Additional Table 1:*** *Variance in repeated measurements of systolic blood pressure.*

***Additional Table 2:*** *Variance in repeated measurements of diastolic blood pressure.*

***Additional Table 3:*** *Variance in repeated measurements of mean arterial blood pressure.*

***Additional Table 4:*** *Effects of different ventilation modes on hemodynamics.*

***Additional Table 5:*** *Variance in repeated measurements of peak airway pressure.*

***Additional Table 6:*** *Variance in repeated measurements of platform airway pressure.*

***Additional Table 7:*** *Variance in repeated measurements of the driving pressure.*

***Additional Table 8:*** *Variance in repeated measurements of central venous pressure.*

***Additional Table 9:*** *Multiple linear regression analysis of central venous pressure and ventilation time.*

***Additional Table 10:*** *Variance in repeated measurements of central venous oxygen saturation.*

***Additional Table 11:*** *Multiple linear regression analysis of central venous oxygen saturation and ventilation time.*

***Additional Table 12:*** *Variance in repeated measurements of heart rate.*

***Additional Table 13:*** *Variance in repeated measurements of nitric oxide.*

***Additional Table 14:*** *Variance in repeated measurements of endothelin-1.*

***Additional Table 15:*** *Variance in repeated measurements of caveolin-1.*

***Additional Figure 1:*** *Effects of different ventilation modes on systolic blood pressure (SBP; A), diastolic blood pressure (DBP; B), and mean arterial blood pressure (MABP; C).*

Additional Figure 1 Effect of different ventilation modes on systolic blood pressure (SBP, A), diastolic blood pressure (DBP, B), and mean arterial blood pressure (MABP, C).
Data are shown as mean ± SD. ^*^P < 0.05, *vs*. 0 hours (repeated measures analysis of variance followed by Bonferroni method).

***Additional Figure 2:*** *Effects of different ventilation modes on peak airway pressure (Ppeak; A), platform airway pressure (Pplat; B) and driving pressure (ΔP; C).*

Additional Figure 2 Effect of different ventilation mode on peak airway pressure (Ppeak, A), platform airway pressure (Pplat, B) and driving pressure (△P, C).
Data are shown as mean ± SD. ^*^P < 0.05, *vs*. 0 hours (repeated measures analysis of variance followed by Bonferroni method).

## Data Availability

*Sequence data supporting the results reported here are available from ResMan Research Manager under the registration number ChiCTR2100043945. Additional data are included within the additional files.*
